# Comparative Analysis of Turmeric and Chlorhexidine Mouthwashes as Anti-plaque and Anti-gingivitis Agents: An Observational Study

**DOI:** 10.7759/cureus.70910

**Published:** 2024-10-05

**Authors:** Zaby Fatima, Ellora Madan, Swati Agarwal, Kratee Sharma, Sheeba Khan, Parv Agarwal

**Affiliations:** 1 Department of Periodontics, Kothiwal Dental College and Research Centre, Moradabad, IND

**Keywords:** gingivitis, mouthwash, plaque, retrospective, stain

## Abstract

Introduction: Mouthwashes are highly efficacious chemical agents for managing plaque and gingivitis. The aim of the present study was to evaluate and compare the efficacy of turmeric (TM) and chlorhexidine (CHX) mouthwash as anti-plaque and anti-gingivitis agents.

Materials and methods: This retrospective, observational study was conducted on the clinical case records of 60 patients with gingival and plaque scores of ≥ 1, who were treated with TM mouthwash as group 1 and CHX mouthwash as group 2 for a period of two months. Indices such as plaque index (PI) and gingival index (GI) were noted at baseline (T0) and at the end of two months of mouthwash use (T1). The data were subjected to statistical analysis.

Results: Both groups were effective in reducing all indices from T0 to T1; however, group 2 was more effective than group 1 in reducing plaque scores at T1. The results were statistically significant (p<0.05), whereas there was no significant difference in the gingival scores at T1 between both groups (p>0.05). Multivariate analysis of variance (MANOVA) revealed no significant differences between the groups in gingival and plaque scores over time. Wilks’ Lambda test revealed that the treatment groups had a statistically significant impact on the overall treatment outcomes.

Conclusion: Although both CHX and TM mouthwashes were equally effective as anti-plaque and anti-gingivitis agents, TM mouthwash is recommended in cases where prolonged use is required, such as in orthodontic patients.

## Introduction

Gingivitis associated with dental plaque represents a reversible inflammatory disorder induced by the accumulation and sustained presence of microbial biofilms on tooth surfaces. It is distinguished by erythema and edema of the gingivae, along with a propensity for the gingivae to exhibit bleeding with minimal provocation [1]. Chlorhexidine digluconate (CHX) is acknowledged as the gold standard for chemical plaque control. It is generally accepted that the mode of action of chlorhexidine is dependent on its initial adsorption to surfaces. Although CHX has a relatively low toxicity following oral administration, it has no side effects. The most common side effects are staining of the tooth surfaces and taste alteration, while less common side effects are dryness, bitter taste, soreness, burning sensation, swelling of salivary glands, mucosal erosions, and numbness. These side effects may negatively influence patient compliance [2, 3]. Staining is the most common problem associated with the long-term use of CHX mouthwash because of non-enzymatic browning (Maillard reaction) and the generation of pigmented metal sulfide compounds within the pellicle [4].

Therefore, several investigations have been conducted on natural compounds such as turmeric (TM) [5, 6]. Turmeric, a rhizomatous herbaceous perennial plant belonging to the ginger family, possesses a remarkable array of biological activities that encompass, but are not limited to, anti-inflammatory properties, antimicrobial effects, and the capacity to act as a potent antioxidant agent, thereby contributing to its widely recognized therapeutic potential in various health conditions. Furthermore, the lipophilic characteristics inherent to the molecular structure of TM enable swift and efficient permeability across cellular membranes, facilitating the bioavailability of its active compounds and enhancing their pharmacological efficacy in the human body. Previous studies comparing TM mouthwash with CHX have reported a significant reduction in plaque, gingival, and gingival bleeding index scores [6, 7].

In a systematic review by Al-Maweri et al., it was concluded that TM mouthwash was as effective in plaque control and gingivitis as CHX mouthwash. Six randomized controlled trials (RCTs) were included; however, all trials were conducted for a maximum period of 28 days [8]. Given the scarcity of extensive long-term studies on this subject, the current investigation was undertaken to evaluate the effectiveness of TM mouthwash in comparison to CHX mouthwash in the management of dental plaque and gingivitis. The secondary objective was to evaluate the effectiveness of both types of mouthwash from the initial measurement to two months of mouthwash application.

## Materials and methods

Study design

This observational, retrospective study was conducted in the Department of Periodontology, Kothiwal Dental College and Research Centre, Moradabad, India, using the records of patients who visited the department between July 2021 and October 2023. Written informed consent was obtained from the department’s protocol to use the records of the patients for research purposes and to maintain confidentiality. The procedure followed in the study was following the ethical standards of the Institutional Ethics and Review Board (approval number: KDCRC/IERB/12/2023/16) and the principles of the Declaration of Helsinki. 

Sample size estimation

The sample size was estimated using G*Power software (version 3.6.9, Heinrich-Heine-Universität Düsseldorf, Düsseldorf, Germany). The present study included a total sample of 60 patients at a power of 80% and an alpha error of 5%. The effect size of 0.64 was taken from a previous study by Waghmare et al. [9]. A mean difference of 0.34 between the plaque index (PI) of CHX and TM mouthwash and a pooled standard deviation (SD) of 0.6 was utilized for calculation.

Eligibility criteria 

This study included the case history records of systemically healthy patients between the ages of 20 and 40 years with gingivitis as diagnosed by clinical examination using a gingival index (GI) by Loe and Silness [10] with scores ≥ 1, PI scores ≥ 1 [11], who were prescribed TM or CHX mouthwash for a minimum of two months, without a history of periodontal therapy within the preceding six months, non-smokers, non-tobacco chewers, and who had complete clinical records with a follow-up of two months. Incomplete records, records with a history of patients using antibiotics, steroids, hormonal therapy within the last six months, multiple missing teeth, periodontally compromised teeth, medically compromised patients, and individuals with disease severity necessitating periodontal therapy were excluded from the study.

Methodology

The records were divided into two groups: group 1, where TM mouthwash was used for gingivitis (n = 30); and group 2, where CHX mouthwash was used (n = 30). Following the comprehensive oral prophylaxis procedure, participants in group 1 utilized a 0.1% TM mouthwash, administered 10 ml for 60 seconds, twice daily, after a 30-minute interval post tooth brushing, and were further advised to refrain from ingestion of food and beverages for 30 minutes following the application of the mouthwash for a duration of two months. No changes were made to the patient’s oral hygiene methods. The patients in group 2 used 0.2% CHX mouthwash following the same instructions and methods as group 1. The following indices were assessed to evaluate the efficacy of two mouth rinses: PI by Turesky et al., who modified Quigley-Hein PI [11], and GI by Loe and Silness [10]. The baseline indices (PI and GI) were obtained from the patient’s case history records (T0). After two months of mouthwash use, the indices were re-recorded (T1).

Statistical analysis

The dataset for the research was input into Microsoft Excel (Microsoft Corp., Redmond, WA) and subjected to analysis using the IBM SPSS Statistics software (IBM Corp., Armonk, NY). Categorical variables, such as sex and frequency of brushing, were expressed in terms of frequencies and percentages, and analysis was conducted using the chi-square test. Continuous variables, including age and clinical parameters (PI and GI), were reported as means and standard deviations, and group comparisons were performed using t-tests. Paired t-tests were used to evaluate alterations between pre-and post-treatment measurements within groups. Inter-group differences and temporal changes were assessed using multivariate analysis of variance (MANOVA). The subsequent analysis utilized the Wilks’ lambda test. A significance level of 0.05 was established as the criterion for statistical relevance.

## Results

The demographic details of the study showed that the participants were evenly distributed between both groups, with 30 (50%) patients in each group. The majority of participants brushed once daily, with 21 (35%) patients in group 1 and 23 (38%) patients in group 2, whereas only 16 (27%) patients brushed twice daily. In group 1, males and females were equally represented, whereas group 2 had a higher proportion of males than females. Overall, 36 (60%) patients were males, and 24 (40%) patients were females (Table 1).

**Table 1:** Demographic details of the study groups

Variables	Type	Group 1		Group 2		Total	
		n	%	n	%	n	%
Brushing	Once	21	35%	23	38%	44	73%
	Twice	9	15%	7	12%	16	27%
	Total	30	50%	30	50%	60	100%
Gender	Male	15	25%	21	35%	36	60%
	Female	15	25%	9	15%	24	40%
	Total	30	50%	30	50%	60	100%

The study compared the PI and GI between both groups over time using an independent t-test. The mean age of the participants was similar in both groups, with no statistically significant difference (p = 0.349). At T0, the PI was comparable between the groups (p = 0.223), showing that the two groups were comparable with respect to baseline data; however, at T1, group 2 showed a significantly greater reduction in plaque scores (1.21 ± 0.44) compared to group 1 (0.71 ± 0.42), with a p-value of 0.001. For GI, no significant differences were observed between the groups at T0 (p = 0.564) and T1 (p = 0.625), with similar reductions in gingivitis scores in both groups (p = 0.465). These results suggest that CHX had a stronger effect on plaque reduction, while gingival outcomes were similar for both CHX and TM mouthwash (Table 2).

**Table 2:** Comparison of plaque index (PI), gingival index (GI), and age between study groups at baseline (T0) and after two months (T1) using the independent T test. *p value ≤ 0.05: significant; CI: confidence interval Data are presented as mean ± standard deviation (SD).

Parameters	Interval	Group 1		Group 2		p-value
		Mean ± SD	95% CI (lower limit-upper limit)	Mean ± SD	95% CI (lower limit-upper limit)	
Age (in years)	Age	34.43 ± 7.05	31.8 - 37.07	32.83 ± 6.05	30.58 - 35.09	0.349
PI	T0	2.21 ± 0.44	2.04 - 2.37	2.35 ± 0.44	2.18 - 2.51	0.223
	T1	1.5 ± 0.27	1.4 - 1.60	1.14 ± 0.30	1.02 - 1.25	0.001*
	Mean difference	0.71 ± 0.42	0.55 - 0.87	1.21 ± 0.44	1.04 - 1.37	0.001*
GI	T0	1.79 ± 0.52	1.6 - 1.99	1.89 ± 0.75	1.61 - 2.17	0.564
	T1	0.97 ± 0.34	0.84 - 1.10	0.93 ± 0.19	0.86 - 1.01	0.625
	Mean difference	0.82 ± 0.67	0.57 - 1.07	0.95 ± 0.70	0.69 - 1.22	0.465

The paired t-test results showed significant reductions in both PI and GI within both groups. In group 2, the PI decreased from 2.35 ± 0.44 (T0) to 1.14 ± 0.30 (T1) (p = 0.001). Similarly, group 1 reported a statistically significant reduction in PI from 2.21 ± 0.44 to 1.5 ± 0.27 (p = 0.001). For the GI, group 2 showed improved gingival scores from 1.89 ± 0.75 to 0.93 ± 0.19, and group 1 showed improvement in gingival scores from 1.79 ± 0.52 to 0.97 ± 0.34, both with significant p-values (0.001), as shown in Table 3.

**Table 3:** Comparison of plaque index (PI) and gingival index (GI) at baseline (T0) and at the end of two months (T1) within groups using the paired T test. *p value ≤ 0.05: significant; CI: confidence interval Data are presented as mean ± standard deviation (SD).

Parameters	Interval	Group 2		Group 1	
		Mean ± SD	95% CI (lower limit-upper limit)	Mean ± SD	95% CI (lower limit-upper limit)
PI	T0	2.21 ± 0.44	2.04 - 2.37	2.35 ± 0.44	2.18 - 2.51
	T1	1.5 ± 0.27	1.4 - 1.6	1.14 ± 0.3	1.02 - 1.25
	p-value	0.001*		0.001*	
GI	T0	1.79 ± 0.52	1.6 - 1.99	1.89 ± 0.75	1.61 - 2.17
	T1	0.97 ± 0.34	0.84 - 1.1	0.93 ± 0.19	0.86 - 1.01
	p-value	0.001*		0.001*	

The MANOVA revealed significant changes in both PI and GI over time. For PI, there was a significant reduction from T0 to T1 (F = 298.19, p = 0.001, η² = 0.6); however, no significant differences were noted between the groups (F = 1.95, p = 0.168). For GI, the reduction over time was statistically significant (F = 100.16, p = 0.001, η² = 0.45), with no significant group effect (p = 0.742) or interaction (p = 0.462) (Table 4).

**Table 4:** Multivariate analysis of variance (MANOVA) for dependent factors df: degree of freedom; η^2^: eta-squared; *p-value ≤ 0.05: significant; RM: repeated measures

Group 1 vs. group 2		Sum of squares	df	Mean square	F value	p-value	η^2^
Plaque index	T0 vs. T1	27.55	1	27.55	298.19	0.001*	0.6
	Group	0.36	1	0.36	1.95	0.168	0.01
	RM factor x group	1.87	1	1.87	20.24	0.001*	0.04
Gingival index	T0 vs. T1	23.7	1	23.7	100.16	0.001*	0.45
	Group	0.03	1	0.03	0.11	0.742	0
	RM factor x group	0.13	1	0.13	0.55	0.462	0

Wilks' lambda analysis assessed the combined effect of treatment (group 1 vs. group 2) on PI and GI over time. The intercept showed a highly significant effect (F = 1092.113, Wilks' Λ = 0.012, p < 0.001), indicating a strong overall change in the dependent variables (PI and GI). The group effect was also statistically significant (F = 8.45, Wilks' Λ = 0.619, p < 0.001), suggesting a meaningful difference between the groups when both indices were considered together across time points. This indicated that the treatment groups had a statistically significant impact on the overall treatment outcomes (Table 5).

**Table 5:** Wilks’ lambda (⸹) analysis for combined effect df: degree of freedom; Num: numerator; Den: denominator; *p-value ≤ 0.05: significant

Cases	df	Approximately F	Wilks' ⸹	Num df	Den df	p
Intercept	1	1092.113	0.012	4	55	0.001*
Group	1	8.45	0.619	4	55	0.001*

## Discussion

The present study was conducted to assess the efficacy of TM and CHX mouthwashes as anti-plaque agents and for the treatment of gingivitis. All studies conducted on this topic were conducted for a short duration (maximum of 28 days) [8]. Therefore, this study was conducted over two months, as CHX is usually recommended for a short duration [2].

The results of the present study indicate that both mouthwashes were effective in reducing gingival and plaque scores from baseline to two months of treatment. This finding is in agreement with previous studies [7-9]. As an antimicrobial oral rinse, CHX demonstrates significant efficacy against a range of pathogens, including bacteria, fungi, and viruses, associated with various oral health conditions. In vitro research has suggested that the antibacterial activity of CHX is associated with alterations in cellular membrane permeability. At higher concentrations (>0.1%), CHX promotes the efflux of vital intracellular components, resulting in bactericidal effects characterized by cellular lysis and subsequent cell death [12]. Chlorhexidine may confer specific clinical benefits in the treatment of gingivitis, as indicated by a systematic review that found that a regimen of daily rinsing with 0.2% CHX for four to six weeks resulted in a significant decrease in clinical symptoms across numerous studies [13]. However, the latest consensus recommendations from the European Federation of Periodontology (EFP) explicitly state that such antiseptic agents should be employed as adjuncts to mechanical tooth brushing and interdental cleaning practices. Turmeric, a polyphenolic entity, is a secondary metabolite extracted from the rhizomes of *Curcuma longa* and has various therapeutic properties, notably antibacterial action. Mohammed et al. [14] demonstrated that the antimicrobial efficacy of TM could be advantageous in the management of dental biofilms. Furthermore, Waghmare et al. [9] indicated a considerable decrease in the overall microbial load with the application of turmeric mouthwash, thus positioning it as a viable option as an anti-plaque agent.

It was observed in our study that at two months, CHX was more effective as an anti-plaque agent than the TM mouthwash; however, it was equally effective as an anti-gingivitis agent. This is in accordance with a study by Chatterjee et al. [7]. The observed decrease in plaque levels during the two-month follow-up interval may be ascribed to the bactericidal effects inherent in CHX and TM mouthwash. The findings from the experimental mouthwash cohort can be primarily explained through its anti-inflammatory characteristics, which impede the activation of nuclear factor kappa-light-chain-enhancer of activated B cells (NF-κB) reliant on tumor necrosis factor (TNF), thereby diminishing the synthesis of reactive oxygen species (ROS). Additionally, TM is recognized for its immunomodulatory functions, which downregulate the expression of the cyclooxygenase-2 enzyme while inhibiting pro-inflammatory enzyme expression. Turmeric also contributes to the downregulation of an array of inflammatory cytokines, including TNF, interleukin (IL)-1, IL-6, IL-8, interferon, and several other chemokines. Similarly, CHX may exert direct anti-inflammatory effects through modulation of the immune response, potentially by attenuating the production of pro-inflammatory cytokines, such as IL-1β and TNF-α, which are integral to the inflammatory cascade.

The MANOVA analysis revealed that over two months, both types of mouthwash were equally effective as anti-plaque and anti-gingivitis agents. This was supported by Muglikar et al. [5], Mali et al. [6], and Waghmare et al. [9], who reported similar anti-inflammatory effects and reductions in microbial counts by TM and CHX. However, our findings contradict those of Malekzadeh et al. [15], who reported TM to be more effective than CHX mouthwash. The reason for the disparity might be that Malekzadeh et al. used oral nano-curcumin as a mouthwash for 28 days.

Wilks’ lambda test in our study revealed that both the treatment groups had a statistically significant impact on the overall treatment outcomes. However, the prolonged application of the CHX mouthwash, extending beyond multiple weeks, can lead to the occurrence of staining, which is ascribed to non-enzymatic browning (Maillard reaction) and the subsequent generation of pigmented metal sulfide within the pellicle. Consequently, this process may enhance the binding interactions between tin and iron with dietary aldehydes and ketones, thereby fostering the accumulation of food components on dental surfaces [12]. Furthermore, other adverse effects associated with the application of CHX mouthwash include xerostomia, alterations in gustatory perception, manifestation of a discolored or coated tongue, burning sensations, and desquamation of the oral mucosa [12]. Therefore, the use of CHX mouthwash is generally discouraged for extended durations, such as during orthodontic treatment, which typically spans two-three years. In contrast, these side effects were not observed with TM mouthwash, making it a preferable option for long-term usage. TM mouthwash is considered safe by the Food and Drug Administration and is not associated with problems of burning sensation or oral desquamation [15].

Clinical implications

Despite its efficacy, CHX mouthwash is associated with adverse effects, including dental discoloration, changes in taste perception, and irritation of the mucosal membranes. In contrast, TM mouthwash has demonstrated comparable effectiveness as an anti-plaque and anti-gingivitis agent, accompanied by a reduced incidence of side effects, thus representing a safer and more patient-centered alternative. Additionally, TM is derived from herbal sources, which confer significant biocompatibility, making it an appealing option for those who prefer natural or biocompatible products over synthetic alternatives such as CHX. Turmeric mouthwash is recommended for extended use in patients undergoing orthodontic treatment.

Limitations

The principal constraint of the current investigation was its retrospective framework, which inherently restricts the applicability of the findings. Moreover, the microbiologic effects of the mouthwashes were not evaluated in our study. Archived data may demonstrate deficiencies or inconsistencies owing to inaccuracies in documentation methodologies or disparities in the approaches utilized for data acquisition. Nevertheless, this issue was meticulously addressed in this study by excluding records with absent or incomplete information. Furthermore, the interactive influence of TM and CHX could not be assessed within the scope of this study. Additional randomized controlled trials (RCTs) are required to validate these long-term implications and comparative advantages.

## Conclusions

The findings of the current investigation demonstrated that both TM and CHX mouthwashes were efficacious in reducing plaque and gingival indices from the baseline to the two-month mark of usage. At the two-month assessment, CHX mouthwash exhibited superior efficacy compared to TM as an anti-plaque agent, while both types of mouthwash displayed comparable effectiveness to anti-gingivitis agents at the same time point. Moreover, the study further elucidated that both mouthwashes maintained equivalent effectiveness in reducing plaque and gingival indices over the duration of the study. The use of TM and CHX mouthwash had a statistically significant impact on the overall treatment outcomes. For extended usage, TM mouthwash is advocated over CHX owing to the inherent complications associated with the latter.
